# Effect of perinatal asphyxia and carbamazepine treatment on cortical dopamine and DOPAC levels

**DOI:** 10.1186/s12929-015-0117-3

**Published:** 2015-02-13

**Authors:** Silvia J López-Pérez, Alberto Morales-Villagrán, Laura Medina-Ceja

**Affiliations:** Laboratorio de Neurofisiología y Neuroquímica, Departamento de Biología Celular y Molecular, CUCBA, Universidad de Guadalajara, Camino Ing. Ramón Padilla Sánchez #2100, Predio Las Agujas, Zapopan, Jalisco C.P 44600 Mexico

**Keywords:** Perinatal asphyxia, Carbamazepine, Dopamine, Frontal cortex, Microdialysis

## Abstract

**Background:**

One of the most important manifestations of perinatal asphyxia is the occurrence of seizures, which are treated with antiepileptic drugs, such as carbamazepine. These early seizures, combined with pharmacological treatments, may influence the development of dopaminergic neurotransmission in the frontal cortex. This study aimed to determine the extracellular levels of dopamine and its main metabolite DOPAC in 30-day-old rats that had been asphyxiated for 45 min in a low (8%) oxygen chamber at a perinatal age and treated with daily doses of carbamazepine. Quantifications were performed using microdialysis coupled to a high-performance liquid chromatography (HPLC) system in basal conditions and following the use of the chemical stimulus.

**Results:**

Significant decreases in basal and stimulated extracellular dopamine and DOPAC content were observed in the frontal cortex of the asphyxiated group, and these decreases were partially recovered in the animals administered daily doses of carbamazepine. Greater basal dopamine concentrations were also observed as an independent effect of carbamazepine.

**Conclusions:**

Perinatal asphyxia plus carbamazepine affects extracellular levels of dopamine and DOPAC in the frontal cortex and stimulated the release of dopamine, which provides evidence for the altered availability of dopamine in cortical brain areas during brain development.

## Background

Perinatal asphyxia (PA) is a principal cause of long-term neurological disability [1], and its severity appears to depend on the intensity of the initial asphyxial event (within the moderate to severe in range). PA can produce secondary brain damage related to the development of attention deficit hyperactivity disorder (ADHD) [2,3] and schizophrenia [4], and both of these diseases are related to altered dopaminergic neurotransmission [5,6]. Seizures are a common response to primary insult that remain after the neonatal period [7-9] and are treated with antiepileptic drugs (AED), such as carbamazepine (CBZ) [10-12], to avoid the consolidation of the epileptic focus. Multiple studies have investigated the effects of PA on the cellular environment, behavior and development [13-21]; however, little is known about the neurochemical brain response to the combination of PA and long-term antiepileptic medical treatment. CBZ has a tricyclic structure [22] and belongs to the first generation of anticonvulsive drugs. The use of CBZ is considered a good treatment choice for various types of epilepsy due to its effectiveness and high tolerance. CBZ has also been used with varying results in the treatment of neuropathic pain [23] and psychiatric disorders [24]. CBZ interacts with different types of membrane receptors and voltage-dependent sodium channels [11] and, consequently, prevents synchronized high-frequency firing [25]. Another effect of CBZ is mediated through an interaction with voltage-sensitive Ca^2+^ channels that influence glutamate release in hippocampus [26].

Dopamine (DA) is a principal catecholaminergic neurotransmitter that is synthesized by tyrosine hydroxylase (TH) and aromatic amino acid decarboxylase [27], activates post-synaptic D1 and D2-type receptors, and is degraded by monoamino-oxidase to 3,4-dihydroxyphenylacetic acid (DOPAC) or catechol-o-methyl transferase to homovanillic acid (HVA) [28]. Previous evidence suggests that DA release is significantly increased during anoxia in a rat-pup model [29,30] and that DA content is decreased in mesostriatal/mesolimbic homogenates of 4-week-old rats with PA [31]. Decreases in DA levels have also been observed in dialysates from the striata of 3- and 6-month-old rats that have been asphyxiated at birth [16,32]. In the mammal brain, one primary dopaminergic system is the mesocortical pathway, which includes cell bodies in the ventral tegmental area (VTA) and synaptic terminals in the frontal and medial cortical areas. Additionally, the mesocortical pathway is related to working and episodic memory, impulsivity control and mood stabilization [20]. Cognitive impairment, ADHD and schizophrenia-related symptoms are related to this dopaminergic system [33-36].

In the present work, we analyzed basal and stimulated DA and DOPAC extracellular contents in the frontal cortices of 30-day-old rats that had been asphyxiated in the early perinatal period and were receiving chronic CBZ treatment. Significant decreases were observed in basal and stimulated extracellular DA and DOPAC content in the frontal cortices of the asphyxiated group, and a partial recovery was observed in the animals that were receiving daily doses of CBZ. Additionally, CBZ elevated basal DA concentrations in the control animals.

## Methods

### Animals

Pregnant female Wistar rats were maintained in individual cages on a 12:12 h light:dark cycle with *ad libitum* access to water and food. At birth, the litters were adjusted to comprise eight male pups. All pups remained with their mother until postnatal day (PD) 30. The animal procedures were conducted according to the National Regulations on Health Research matters (Mexico) and directive 86/609/EEC for animal experiments. All efforts were made to minimize animal suffering and the number of animals used.

### Experimental groups

At PD11, the pups were assigned to one of the following experimental groups: the control group (without manipulation; the animals remained with their mother the entire time); the CBZ group (received daily doses of 50 mg/kg CBZ dissolved in distilled water via oral administration from PD11 to PD30); the PA group (asphyxiated at PD11); and the PA + CBZ group (asphyxiated at PD11 and treated with daily doses of 50 mg/kg CBZ dissolved in distilled water via oral administration until PD30).

### Asphyxia induction

The PD11 pups assigned to the PA and PA + CBZ groups were placed in small Plexiglas cage (10×10×10 cm) with a sealable lid. An oxygen sensor was coupled to a lateral wall (Maxtec Inc., Salt Lake, USA) and connected through an interface (Pasco Scientific, CA, USA) to a computer. The animals were subjected to asphyxia conditions with an 8% O_2_/92% N_2_ gas mixture for 45 min and then maintained for 15 min in the cage under normoxic conditions (reperfusion period). The pups were subsequently returned to their litter, where they remained until PD30. During the asphyxia period, the pups’ behaviors were recorded by video and subsequently analyzed by a spotter who was blind to the experimental conditions.

### Microdialysis procedure

When the previously asphyxiated animals reached PD30, they were subjected to a stereotaxic surgery to insert a microdialysis probe (CMA7, 7-mm shaft length and 2-mm membrane length, 6-kDa cut off, mean recovery 32 ± 5.6%) into frontal cortex (AP: 3.2 mm, L: 2.8 mm and D: −3 mm relative to bregma under isoflurane anesthesia). Throughout the experiment, the probe was perfused with oxygenated Krebs-Ringer solution (all concentrations are in mM: NaCl 118; KCl 4; KH_2_PO_4_ 1.25; MgSO_4_ 1.17; CaCl_2_ 2.2; NaHCO_3_ 25; and glucose 10). Once the procedure was completed, the anesthesia was removed, and after an equilibration period of 2 h, dialysis fractions were collected (flow rate of 2.5 μl/min, one sample every three min) according to the following experimental protocol: fraction 1–2, basal conditions (Krebs-Ringer solution); fraction 3–4, stimulating conditions (20 mM 4-AP in Krebs-Ringer solution via reverse microdialysis through the same probe with 15 μg of 4-AP measured and dispensed into the brain); and fraction 5–13, return to basal conditions (Krebs-Ringer solution). The fractions were collected in an antioxidant solution (0.4 mM sodium metabisulfite in 0.1 M perchloric acid) and stored at −20°C until the DA and DOPAC content quantifications were performed by HPLC. After each experiment, the animal was deeply anaesthetized with nembutal and transcardially perfused with phosphate buffer (0.1 M PBS, pH 7.4), followed by 4% paraformaldehyde. The brains were obtained and post-fixed for 48 h at 4°C, and 40-μm-thick coronal sections were excised and stained with cresyl violet to ensure the proper location of the guide cannula in right frontal cortex. Animals with incorrectly placed cannulae were excluded from the analyses.

### DA and DOPAC measurements

The samples were analyzed for DA and DOPAC content in a HPLC system using electrochemical detection that consisted of an isocratic pump (Waters Corporation, Milford, MA, U.S.A., Waters 515, flow 0.2 ml/min), an RP-18 column (Waters Corporation, Milford, MA, U.S.A., Xterra, 5 μM particle size, 2.1x100 mm, Waters), and an amperometric detector (BAS Inc., West Lafayette, IN, U.S.A., LC-4B, oxidation potential 0.5 V). The elution medium was composed of 2.2 mM octanesulfonic acid sodium, 14.7 mM sodium phosphate, 30 mM sodium citrate, 0.027 mM EDTA (pH 3.5), and 4% acetonitrile. The amperometric detector signal was digitalized with eDAQ Power Chrome software (eDAQ Inc., Colorado Springs, CO, U.S.A., version 2.6.3). In all of these conditions, the DA and DOPAC elutions were achieved at 4.5 and 7.5 min, respectively, and the limit of detection for both molecules was 1.25 nM.

### Statistical analyses

The basal concentrations of DA and DOPAC are reported in nanomolar concentrations (mean ± SD). Significant differences between the groups were estimated with one-way ANOVA tests, followed by Tukey’s post-tests. The temporal course and stimulated extracellular DA and DOPAC levels are expressed as percentages of the control basal group, for which the mean value of the three first fractions was considered 100%. The differences between the basal and stimulated fractions were estimated using one-way ANOVA tests, followed by Dunnett’s post-tests, and the specific differences between groups were estimated via Tukey’s post-tests. In all cases, statistical significance was considered at p < 0.05, and the tests were performed with the GraphPad Prism software, version 5.00, for Windows (GraphPad Software, San Diego CA, U.S.A.).

## Results

### Behavioral observations during the induction of asphyxia

During the asphyxia procedure, the pups exhibited the following behaviors 2–3 min after asphyxia onset: gasping, sudden jumps, repetitive head tremors, and forepaw automatisms. By the end of asphyxia period, the pups were cold and had pale skin colorations compared with the control animals. The temperature and skin color were recovered in the following 4–5 hours. In contrast, the control pups placed in the same cage for the same period of time exhibited quiet constant breathing, normal temperature and skin color and no indications of any of the asphyxia behaviors described above.

### Extracellular measurements of basal DA and DOPAC

**Figure 1:**
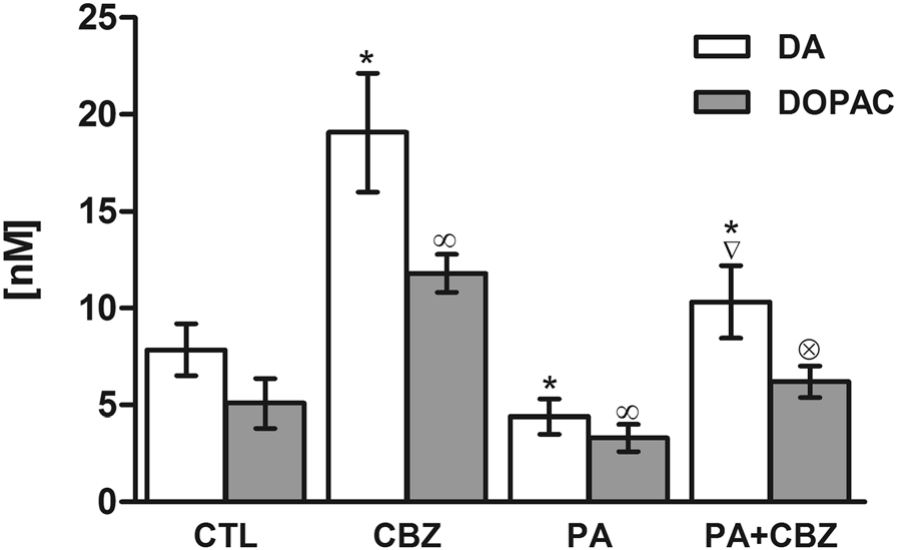
**Basal extracellular levels of DA and DOPAC.** The measurements were made on PD30 from rats that had been asphyxiated at PD11 in the control group (CTL), carbamazepine-treated group (CBZ), asphyxiated group (PA) and asphyxiated plus carbamazepine-treated group (PA + CBZ). *p < 0.05 vs. CTL for DA; ∞p < 0.05 vs. CTL for DOPAC. ▽p < 0.05 PA + CBZ group vs. PA group for DA; ⊗p < 0.05 PA + CBZ group vs. PA group for DOPAC.

The basal extracellular DA and DOPAC levels measured in the control groups were 7.85 ± 1.35 nM and 5.09 ± 1.28 nM (n = 12), respectively. Compared with the control group, the CBZ-treated group exhibited significant increases in DA (7.85 ± 1.35 nM for CTL vs. 19.07 ± 3.06 nM for CBZ) and DOPAC content (5.09 ± 1.28 nM for CTL vs. 11.81 ± 0.97 nM for CBZ), whereas PA decreased DA (4.40 ± 0.89 nM for PA) and DOPAC (3.31 ± 0.69 nM for PA) content. Finally, the pups that were asphyxiated and treated with CBZ exhibited recoveries of extracellular DA (10.33 ± 1.87 nM) and DOPAC (6.20 ± 0.81 nM) to slightly higher levels than those of the control group (Figure 1).

### Extracellular measurement of stimulated DA and DOPAC

**Figure 2:**
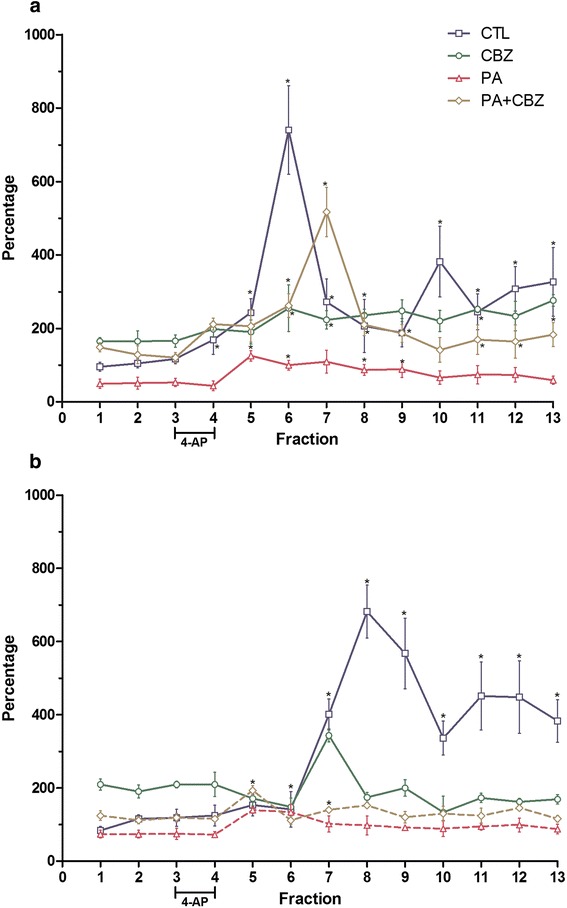
**Temporal courses of DA in (a) and DOPAC (b).** Microdialysates were obtained at PD30 from rats that had been asphyxiated on PD11 in the control group (CTL), carbamazepine-treated group (CBZ), asphyxiated group (PA) and asphyxiated plus carbamazepine-treated group (PA + CBZ). Each fraction represents a 3 min sample, and the values are presented as percentages of the basal period of the CTL group, which was calculated by taking the mean of the first three fractions as 100%. *p < 0.05 each fraction vs. its baseline.

To stimulate DA release, a sub-convulsive dose of 4-aminopyridine (4-AP) was administered via a reverse dialysis procedure into frontal cortex. Figure 2a shows the temporal course of DA release. The control group exhibited an expected increase in DA following 4-AP infusion in the tissue, followed by an elevated extracellular concentration for at least the subsequent 20 min. Interestingly, the animals that were chronically treated with CBZ did not exhibit any increase in the extracellular concentration of DA, although the basal levels of this group were higher than that of the control group. However, the animals that had been asphyxiated at PD11 exhibited a temporal profile that involved low extracellular concentrations compared with the control group, whereas the asphyxiated animals that had been chronically treated with CBZ recovered extracellular DA to a level that was significantly higher than that of the PA group (Figure 2a).

In contrast, the control group exhibited the expected 4-AP-induced increase in DOPAC; the DA response was somewhat temporally delayed, and higher levels were observed until the end of collection. The CBZ group exhibited a higher basal level (relative to the control group), and a transitory peak was induced by the 4-AP infusion. The PA group exhibited a significant reduction in extracellular DOPAC concentrations, and a small peak was induced following the infusion of 4-AP; however, no further differences were observed with respect to basal DOPAC levels. Chronic CBZ administration induced a significant elevation in extracellular DOPAC level and a small and transient response to 4-AP that quickly returned to basal levels (Figure 2b).

## Discussion

Asphyxia at birth causes a severe energetic crisis in the brain, which strongly depends on oxygen supply for optimal functioning. Unfortunately, asphyxia is not a fully predictable condition, and identifying strategies for possible delayed complications, such as seizures [37], ADHD and schizophrenia [38], is necessary. The use of hypothermia has been considered a promising strategy and has produced good results with several experimental approaches [1,39]; however, its implementation is not possible in most countries. In contrast, AEDs, such as CBZ, have long been used as the first line of action in the control and prevention of the seizures associated with PA. Previous studies have explained the generation of short-term seizures caused by PA and related oxygen deprivation to early EEG abnormalities [40], the participation of glutamate receptors [41] and high extracellular glutamate levels in the brain tissue during asphyxia [42]. These abnormalities have also been associated with the complex metabolic cascades activated in response to insult to cause ROS production [43] and changes in neurochemical markers [42] and gene expression [44]. Altogether, these alterations may favor the perpetuation of the initial damage and thus promote conditions favorable for subsequent changes to neurotransmitter systems, including the mesocortical dopaminergic system, which is related to the hallmarks of ADHD and schizophrenia. However, in clinical conditions, pediatric patients with PA are subjected to pharmacological management, and the information regarding the neurochemical responses of neurotransmitter systems that are relevant to brain function during and after such pharmacological treatment is insufficient. In this context, this study aimed to analyze basal and stimulated DA and DOPAC extracellular contents in the frontal cortex of 30-day-old rats that had been asphyxiated in the early perinatal period and were receiving chronic CBZ treatment. To our knowledge, there are no previous reports related to the combined effects of PA and CBZ on DA cortical release.

The animals treated with CBZ exhibited increases in basal DA and DOPAC concentrations. These effects contrast with the data available in the literature because CBZ is well known to inhibit voltage-activated sodium channels and thus diminish high-frequency neuronal firing [45]. Additionally, CBZ interferes with L-type calcium currents [26]. These observations suggest that CBZ should reduce the release of neurotransmitters. However, CBZ may not exert such an effect on dopaminergic neurons. Additionally, previous evidence suggests that DA can be released in a calcium-independent manner in both synaptosomes and striatum slices [46,47], even when calcium channels are blocked [48], which accords with the results reported here.

When the basal extracellular DA and DOPAC concentrations were measured in the frontal cortices of the previously asphyxiated animals, concentrations were reduced by approximately 50%, and these reductions reflect an important long-term susceptibility of the dopaminergic system to asphyxia. Directly related to these results, both the Bustamante [49] and Chen [50] groups showed region-specific effects on the basal levels of DA and metabolites in 4-week-old animals born via cesarean section (C-section) and subjected to later immersion in a 37°C water bath. Interestingly, decreased DA and DOPAC levels were observed in the nucleus accumbens, a terminal field of the mesolimbic dopaminergic system, in animals asphyxiated for more than 20 min [50]. When the measurements were repeated at 2–3 months after asphyxia in the same group, significantly reduced striatal DA and DOPAC levels were observed [16]. The reductions in basal cortical extracellular DA and DOPAC levels observed in the present work are consistent with these data. Interestingly, previous studies have shown that the dendritic trees of neurons obtained from asphyxiated animals are smaller [51] and that the structural organizations of the synaptic densities in the striatal tissue of 45-day-old rats asphyxiated at birth are altered [17]. Together, these results suggest that PA can cause alterations that underlie the depletions of basal extracellular DA and DOPAC concentrations observed here and in other studies, despite the differences in the asphyxia induction models used.

When the release of DA was stimulated with 4-AP in the previously asphyxiated rats, the expected elevations in the extracellular concentrations of DA and DOPAC were observed shortly after the 4-AP infusion began. This drug has an antagonistic effect on voltage-gated potassium channels that delays the repolarization phase of the action potential and thus facilitates the synaptic release of neurotransmitters [52]. This drug has been used to produce seizures in experimental animals [53,54] and as a therapy in the treatment of multiple sclerosis [55]. Notably, despite the elevated basal DA concentrations in the animals treated with daily doses of CBZ, the extracellular DA concentrations following 4-AP stimulation were not different from the baseline at any time. In these animals, only DOPAC exhibited a transient increase 9–10 min after 4-AP infusion. CBZ exerts an inhibitory effect on the frequency of neuronal firing [45] that is mediated through a partial block of voltage-gated sodium channels. Moreover, there is evidence that CBZ interferes with calcium-dependent action potentials [26], which may influence receptor function via structural changes caused by altered phosphorylation or subunit compositions; similar (but much more extreme) phenomena have been observed in models of hypoxia/ischemia injury [56-58]. We speculate that these types of cellular mechanisms are operating during CBZ treatment (from PD11 to PD30 here) to alter the stimulated release of DA by 4-AP while maintaining the extracellular concentration of DA without altering the responses to stimuli. These data are relevant for pediatric patients with histories of PA and CBZ treatment for prolonged periods because such histories are likely to interfere with normal brain function, as has been reported by other authors [59,60].

## Conclusion

In conclusion, PA decreased the extracellular content of DA (and its principal metabolite, DOPAC) in the frontal cortex. This release was not fully recovered with CBZ (moreover, despite the higher DA baseline concentration, no increase was observed in the DA response to 4-AP stimuli). The causes of this lack of response require further study. The limited availability of DA in cortical brain areas during brain development is more relevant from the perspective of the wide developmental window of the dopaminergic system because although DA innervation and receptor expression are present from early development onwards, the maturation of this system continues during adolescence, and stable patterns continue to form until maturity; these processes involve cellular and molecular mechanisms that depend to some extent on the presence of DA in the extracellular space.
